# Risk factors for posterior capsule opacification following phacovitrectomy: a six-year retrospective analysis

**DOI:** 10.1007/s10792-026-04017-7

**Published:** 2026-03-11

**Authors:** Mieszko Lachota, Agata Frajdenberg, Karolina Radl Steiner, Wojciech Hautz, Bjorn Johansson, Marcin Piotr Czajka

**Affiliations:** 1 Department of Ophthalmology, Children’s Memorial Health Institute, Warsaw, Poland; 2 Department of Ophthalmology, Department of Clinical Sciences, Linköping University, Linköping, Sweden

**Keywords:** Posterior capsule opacification, Phacovitrectomy, Cataract, IOL

## Abstract

**Purpose:**

To study risk factors for Nd:YAG capsulotomy (YAG-CT) due to visually disturbing posterior capsule opacification (PCO) after combined cataract surgery and vitrectomy, phaco-vitrectomy (PhV).

**Methods:**

Single-center retrospective comparative cohort study. We included 196 patients (197 eyes) undergoing PhV. Electronic medical records provided baseline patient data, data on indication for vitrectomy, surgery data including intra-ocular lens (IOL) type, complications, and YAG-CT incidence during 6-year follow-up after PhV. Univariate and multivariate regression analyses assessed associations of various factors with YAG-CT incidence.

**Results:**

Fifty-four eyes (27.41%) underwent YAG-CT during the follow-up period. One of the three hydrophilic acrylic IOLs showed the lowest YAG-CT incidence. Adjusted Odds Ratios (aORs) for YAG-CT were significantly higher for the hydrophobic acrylic IOL (aOR = 5.85, *p* < .05), and the two other hydrophilic acrylic IOLs (aOR = 29.0, *p* < 0.001 and aOR = 79.4, *p *< 0.001). Compared with PhV for macular hole, PhV for epiretinal membranes (aOR = 9.9, *p* < 0.01), retinal detachment (aOR = 25.4, *p* < 0.01), and silicon oil removal (aOR = 22.4, *p* < 0.05) correlated with higher YAG-CT incidence. Type 2 diabetes correlated with increased YAG-CT incidence (aOR = 6.7, *p* < 0.01).

**Conclusion:**

IOL type is a key factor in development of visually disturbing PCO after phacovitrectomy. One hydrophilic acrylic IOL outperformed other examined IOLs in reducing YAG-CT incidence after PhV. Retinal disease, as well as type 2 diabetes, correlated with YAG-CT, and should motivate a choice of an IOL type associated with a low YAG-CT incidence for PhV.

## Introduction

Phacovitrectomy combines cataract surgery with pars plana vitrectomy. It gained popularity among both surgeons and patients due to improved visualization during vitrectomy and a decreased the number of surgeries needed, lowering the associated costs and total visual recovery time [1]. Recent development of small-gauge instruments and corneal microincisions further improved and popularized this technique [2].

Posterior capsule opacification (PCO), the most common complication after both cataract surgery and phacovitrectomy, occurs when the posterior capsule of the lens becomes opaque due to lens epithelial cells migrating from the equator of the capsular bag behind the optic of the intraocular lens (IOL) implant. PCO symptoms include blurred vision, glare, and decreased contrast sensitivity, all significantly decreasing patient’s visual acuity and quality of life. Treatment for PCO following cataract surgery typically involves a YAG capsulotomy (YAG-CT), which, despite its relative simplicity, is not free of complications [3]. After phacovitrectomy, the complications of YAG-CT have not yet been studied. Compared to cataract surgery, PCO develops earlier and more often after combined surgery, with an incidence rate varying between 10 and 76% [3–10].

Initial attempts at decreasing PCO risk based on capsule polishing aiming at removing all residual lens epithelial cells (LEC) have failed to bring any significant benefit [11]. An effective measure of decreasing PCO incidence is performing a primary posterior capsulotomy [12]. However, it did not gain popularity except in pediatric cataract surgery due to technical challenges and investigators have shifted towards designing IOLs with anti-PCO properties [13].

Both IOL material and shape are being fine-tuned to decrease PCO risk. Most studies report lower PCO rates for hydrophobic acrylic IOLs and IOLs with sharp posterior optic edge [14–20]. However, these studies are limited by comparing IOLs that differ in multiple aspects at a time, e.g., material, edge design, and haptic angulation [14–16, 18–20]. So far, there has been only one randomized clinical trial comparing PCO incidence between IOLs differing in only one design characteristics, where the IOLs were made with the same material and haptic angulation, with only a difference in posterior optic edge design [17]. The lack of other studies with well-designed controls might have led to incorrect conclusions that are currently guiding surgeons in IOL choice [21].

The knowledge about PCO risk factors after phacovitrectomy is scarce. Moreover, knowledge about PCO after cataract surgery cannot be applied for phacovitrectomy patients as novel and important variables such as underlying retinal disease are introduced. After an extensive literature search, we found only one retrospective analysis, which identified factors such as rhegmatogenous retinal detachment, axial length over 24.5 mm, intraoperative/postoperative complications, C2F6 tamponade, and postoperative posturing as significantly associated with a higher risk of PCO development [9]. However, the study was limited by a short observation time (6 months) and univariate analysis, potentially overlooking interrelationships between variables.

Hence, a need to re-evaluate the factors contributing to PCO has emerged. Therefore, we used retrospective data to study the incidence and risk factors of visually disturbing PCO, assessed by the incidence of Nd:YAG capsulotomy, after 23- and 25-gauge phacovitrectomy with implantation of different hydrophobic or hydrophilic acrylic IOLs through a corneal microincision.

## Materials and methods

The study was conducted in accordance with the principles of the Declaration of Helsinki. The Regional Ethical Review Board in Linköping approved the study protocol (registration number M52-08). Register data and patient records for 197 consecutive phacovitrectomy surgeries (196 patients) performed between 2014 and 2015 at the Department of Ophthalmology, Linköping University Hospital, were retrospectively reviewed.

All patients were followed for 72 months after surgery. The surgery was performed by four surgeons (MC,AF,BJ,AW), in local or general anesthesia. Clear corneal incision of 1.8–2.2 mm, a well centered 5 to 6 mm continuous curvilinear capsulorhexis created with microforceps, and implantation of the IOL in the capsule using an injector were performed in all cases. Sutures were not used to close the corneal tunnel incision except in cases with significant leakage from the corneal incision during vitrectomy. Vitreous surgery was performed using a 23-gauge or 25-gauge vitreous cutter driven by a vitrectomy unit and the 1-step trocar system. Infection prophylaxis consisted of rinsing the conjunctival sac of the eye to be operated on with Chlorhexidine solution 0.05%. The skin around both eyes was carefully swabbed with Chlorhexidine alcohol 0.5%. No antibiotic was added to the irrigation fluid. 1 mg Zinacef® (cefuroxim) was injected (0.1 ml of Zinacef® solution 10 mg/ml) into the anterior chamber at the end of the surgery. Additionally, 0.5 ml Zinacef® solution 250 mg/ml was injected subconjunctivally; the volume was divided into three and given in the vicinity of each sclerotomy. Betamethasone was injected subconjunctivally at the end of the surgery. Single topical dexamethasone together with cyclopentolate was used for three weeks postoperatively.

The following data were extracted and transferred to a database: patient age and gender, indications for vitrectomy, type of IOL, type of tamponade, intraoperative and postoperative complications, intraoperative use of triamcinolone, duration of surgery, dates of surgery and-where applicable-date(s) of YAG-CT and/or death. Patients and dates of operation were identified through the electronic cataract operation national register, which provided information on type of surgery (combined). We did not evaluate the axial length of the patient’s eye because of its measuring difficulty in cases of macula-off retinal detachment. Clinical patients records, using Cosmic software system, provided information on phacovitrectomy, YAG-CT and eventual date of patient deaths. Eyes with capsulotomy performed during silicon oil removal after previous phacovitrectomy with silicon oil tamponade were also included in the analysis. Cases were excluded if capsule damage during the surgery precluded secure placement of the IOL in the capsular bag.

The association of different variables with PCO requiring YAG-CT was evaluated through univariate and multivariate logistic regression using “glmnet” and visualized using “forestploter” packages in R. The variables for multivariate logistic regression were chosen using a complete enumeration algorithm with AIC criterion, implemented in “bestglm”. To decrease study bias, multivariate analysis, standardized data collection methods, and transparent method reporting were implemented. No formal calculation was conducted to arrive at the study size. Twenty patients were lost to follow-up, reaching an acceptable 90% follow-up. The date of PCO occurrence was not specified in 14 eyes.

## Results

The average age at the time of PhV was 69 ± 9 years (mean ± SD). During the 6-year follow-up period, YAG-CT was carried out in 54 (27.41%) eyes. YAG-CT was performed within 1 year after the surgery in 5 eyes, between 1 and 3 years in 20 eyes, and later than 3 years in 15 eyes. Timing of YAG-CT in the remaining 14 eyes was not recorded. The clinical characteristics of our cohort are summarized in Table 1.

**Table 1:** Clinical characteristics of our cohort

Characteristics	Our cohort (196 patients/197 eyes)
*Age at diagnosis (years)*	
Mean (SD)	69 (9)
Min–max	28–91
*Gender*	
Female	108 (55.1%)
Male	88 (44.9%)
*PCO*	
Yes	54 (27.41%)
< 12 months	5 (2.54%)
12–36 months	20 (10.15%)
> 36 months	15 (7.61%)
not specified	14 (7.11%)
No	143 (72.59%)
*Diagnosis*	
ERM	57 (28.93%)
RRD	41 (20.81%)
MH	42 (21.32%)
VH	23 (11.68%)
VTM	13 (6.6%)
SOR	10 (5.08%)
TRD	4 (2.03%)
Other*	7 (3.55%)
*IOL*	
MJ14	160 (81.22%)
MI60	17 (8.63%)
PCB00	12 (6.09%)
Asphina 409 M	8 (4.06%)
*Sclerotomy*	
23G	57 (28.93%)
25G	140 (71.07%)
*Tamponade*	
Air	91 (46.19%)
SF6	88 (44.67%)
C2F6	9 (4.57%)
Silicon oil	6 (3.05%)
C3F8	3 (1.52%)
*Triamcinolone*	
No	168 (85.28%)
Yes	29 (14.72%)
*Intraop. complications*	
No	154 (78.17%)
Yes	43 (21.83%)
*Postop. complications*	
No	171 (86.8%)
Yes	26 (13.2%)
*Surgery duration*	
Mean (SD)	79 (37)
Min–max	22–297
*Diabetes*	
No	162 (82.23%)
Type 1	2 (1.02%)
Type 2	33 (16.75%)

ERM, epiretinal membrane; RRD, rhegmatogenous retinal detachment; MH, macular hole; VH, vitreous hemorrhage; VTM, vitreomacular traction; SOR, silicon oil removal; TRD, tractional retinal detachment

*Other indications included amotio retinae, optic nerve pit, Proliferative diabetic retinopathy, submacular hemorrhage and vitreous floaters

**Fig. 1:**
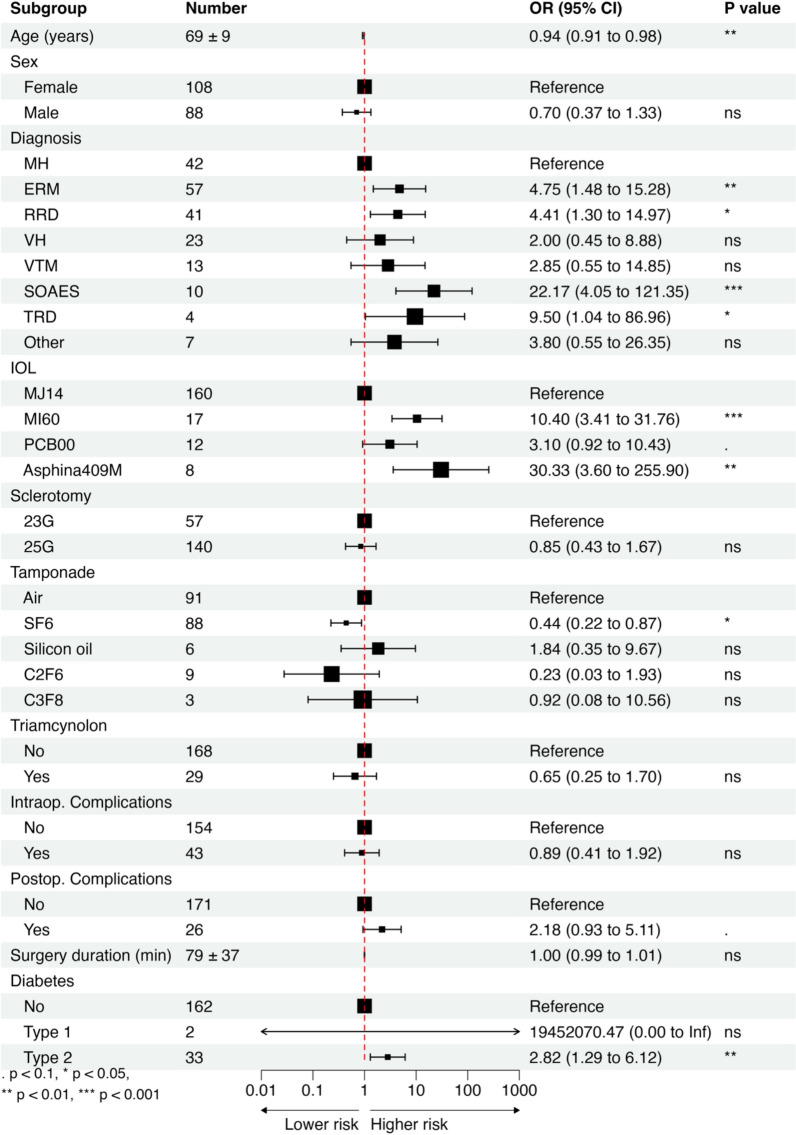
Forest plot of odds ratios (OR) from univariate analysis of risk factors for posterior capsule opacification (PCO). MH, macular hole; ERM, epiretinal membrane; RRD, rhegmatogenous retinal detachment; VH, vitreous hemorrhage; VTM, vitreomacular traction; SOR, silicon oil removal; TRD, tractional retinal detachment. Other indications included amotio retinae, optic nerve pit*,* Proliferative diabetic retinopathy, submacular hemorrhage and vitreous floaters.Statistical significance was assessed by a t-test. Significance thresholds: (***)*p* < 0.001; (**)*p* < 0.01; (*)*p* < 0.05; (.) *p* < 0.1; (ns) *p* ≥ 0.1

**Fig. 2:**
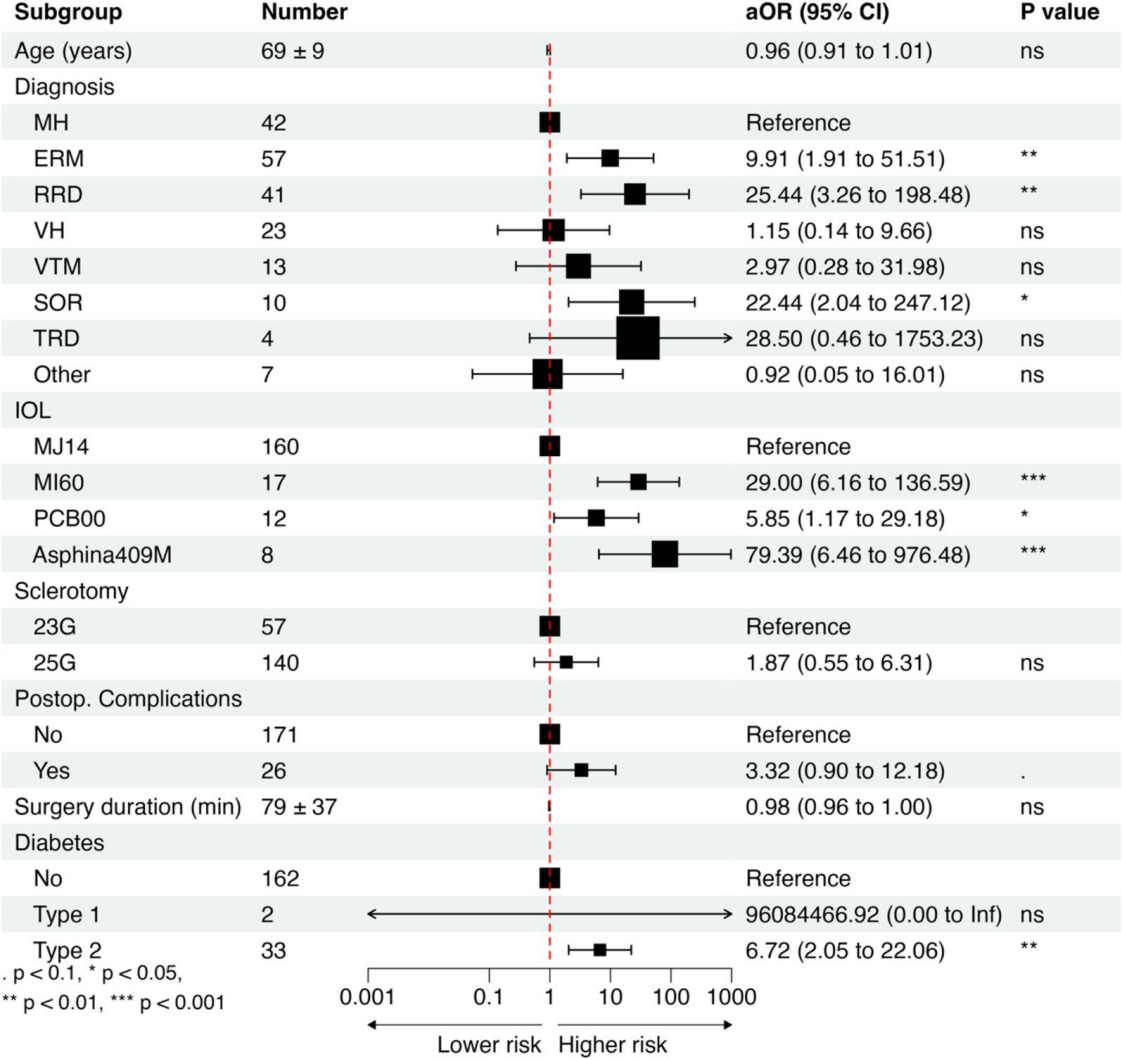
Forest plot of adjusted odds ratios (aOR) from multivariate analysis of risk factors for posterior capsule opacification (PCO). MH, macular hole; ERM, epiretinal membrane; RRD, rhegmatogenous retinal detachment; VH, vitreous hemorrhage; VTM, vitreomacular traction; SOR, silicon oil removal; TRD, tractional retinal detachment.*Other indications included amotio retinae, optic nerve pit*,* Proliferative diabetic retinopathy, submacular hemorrhage and vitreous floaters.Statistical significance was assessed by a *t*-test. Significance thresholds: (***)*p* < 0.001; (**)*p* < 0.01; (*)*p* < 0.05; (.)*p* < 0.1; (ns)*p* ≥ 0.1

In order to identify the key risk factors contributing to visually disturbing PCO development following phacovitrectomy necessitating YAG-CT, we first performed univariate regression analysis to evaluate each variable separately (Fig. 1). Then, we set out to identify the combination of the most important factors contributing to PCO development using a complete enumeration algorithm as described in methods. Age, underlying retinal disease, IOL type, occurrence of postoperative complications, size of sclerotomy, surgery duration, and diabetes were included in the final model in multivariate analysis (Fig. 2).

Indications for vitrectomy are presented in Table 1. For the purpose of the analysis, we used the diagnosis of macular holes (MH) as a reference level. Both univariate (OR = 4.75, *p* < 0.01) and multivariate (aOR = 9.91, *p* < 0.01) analyses showed an increased occurrence of PCO following phacovitrectomy treatment of epiretinal membranes (ERM) compared to MH (Fig. 1 and 2). Rhegmatogenous retinal detachment and removal of silicon oil after previous surgery were also associated with an increased incidence of YAG-CT due to visually disturbing PCO compared to ERM, with aOR = 25.44 (*p* < 0.01) and aOR = 22.44 (*p* < 0.05), respectively (Fig. 2). Tractional retinal detachment was associated with increased YAG-CT incidence. However after adjusting for other variables, the results were not statistically significant. Vitreous hemorrhage and vitreomacular traction were not associated with a higher YAG-CT incidence.

The main characteristics of all lenses implanted during surgery are summarized in Table 2. Compared to MJ14 (Bausch&Lomb), implantation of other lens types carried a 10.4 (*p* < 0.001), 30.33 (*p* < 0.01), 3.1 (*p* < 0.1) times increased risk of YAG-CT due to visually disturbing PCO for MI60 (Bausch & Lomb), Asphina 409 M (Zeiss), and PCB00 (J&J), respectively (Fig. 1). Adjusted for other factors, implantation of IOLs other than MJ14 carried a 29.0 (*p* < 0.001), 79.39 (*p* < 0.001), 5.85 (*p* < 0.05) times increased YAG-CT incidence for MI60, Asphina 409 M, and PCB00, respectively (Fig. 2).

**Table 2:** Properties of evaluated intraocular lenses

	MJ14 Incise	Akreos MI60	PCB00	Asphina 409M
Manufacturer	Bausch & Lomb, Bridgewater, NJ	Bausch & Lomb Bridgewater, NJ	Johnson & Johnson, New Brunswick, NJ	Carl Zeiss Meditec, Jena, Germany
Type	Monofocal, aspheric	Monofocal, aspheric	Monofocal, aspheric	Monofocal, aspheric
Material	Hydrophilic acrylic (22% H_2_O)	Hydrophilic acrylic (26% H_2_O)	Hydrophobic acrylic	Hydrophilic acrylic (25% H_2_O) with hydrophobic surface properties
Edge	360° Barrier posterior optic edge, curvature radius 5 μm	360° Posterior square edge	360° posterior square edge, ProTEC frosted	360° Square edge
Incision size (suggested)	1.4^*^/1.8 mm	1.8 mm	2.2–2.4 mm	1.8 mm
Lens design	One-piece IOL with four-point fixation	One-piece IOL with four-point fixation	One-piece IOL with two-point fixation	One-piece MICS with four-point fixation
Haptic angulation	3°–10° across dioptre range	10°	0°, haptics offset anteriorly to posterior optic surface	0°
A-constant (SRK-T, suggested)	118.4	119.1	119.3	118.0
Optic diameter	5.6–6.2 mm across dioptre range	5.6–6.2 mm across dioptre range	6.0 mm	6.0 mm
Total diameter	11.0 mm	10.5–11.0 mm	13.0 mm	11.0 mm
UV filter	Yes	Yes	Yes	No

*wound-assisted technique

In our cohort, every year of age was associated with a 0.94 (*p* < 0.01) decrease in YAG-CT rate in univariate analysis. However, the effects of older age were not statistically significant when corrected for other variables (*p* = 0.13).

Type 2 diabetes was associated with a higher YAG-CT incidence, aOR = 6.72 (*p* < 0.01). The results were not statistically significant in type 1 diabetes. However, we noted that all the patients with T1D (N = 2) had YAG-CT due to visually disturbing PCO (Fig. 1 and 2) during the study period.

Gas tamponade with SF6 was associated with a lower YAG-CT incidence compared to an air tamponade in univariate analysis (OR = 0.44, *p* < 0.05, Fig. 1). When corrected for other variables, the type of tamponading agent had no effect on YAG-CT incidence.

Surgery duration and sclerotomy size were not significant factors affecting YAG-CT incidence although they were included in multivariate analysis to improve the predictive power of the model. Intraoperative complications, triamcinolone use, and sex of the patient did not correlate with YAG-CT incidence (Figs. 1 and 2).

## Discussion

We retrospectively evaluated risk factors for YAG-CT performed due to visually impairing PCO following phacovitrectomy in a 72-month follow-up. Our study indicates that IOL type, the underlying retinal disease, as well as type 2 diabetes have considerable impact on PCO formation. Establishing these risk factors may help to predict the risk of PCO development and, most importantly, allow to decrease PCO incidence by acting through the identified modifiable risk factors, e.g., by choosing an optimal IOL.

Due to the clinical failure of both LEC removal and pharmacological intervention in mitigating PCO, attention has shifted to IOL design as a future solution [11, 22]. The implanted IOL type is established as the most important modifiable factor determining PCO occurrence after cataract surgery. Both IOL material and design have been shown to play critical roles for PCO formation and YAG-CT rate [13, 17, 20, 23, 24]. The IOL materials’ influence is likely due to the different binding properties of extracellular matrix proteins including fibronectin and vitronectin, which promote lens epithelial cell migration and growth [25]. Recent studies on IOL materials report that acrylic lenses exhibit considerably lower rates of PCO compared to polymethylmethacrylate (PMMA) and silicone lenses [18, 20]. Additionally, hydrophobic acrylic IOLs were considered to be superior to hydrophilic acrylic IOLs in reducing PCO formation after cataract surgery [18, 19]. With respect to IOL design, the proper design of a sharp posterior optic edge plays a pivotal role in preventing PCO formation by acting as a mechanical barrier, as demonstrated by lower PCO incidence of IOLs sharp edges compared with round edge IOLs as well as lower PCO rates with plano-convex optic compared to biconvex [14–16].

Similarly to cataract surgery, our study demonstrated that IOL is the key modifiable PCO risk factor following phacovitrectomy. Beneficial anti-PCO properties of MJ14 IOL are present independently of underlying retinal disease, sclerotomy size, postoperative complications, surgery duration, age or diabetes, indicating its superior performance in all groups of patients.

Contrary to studies suggesting the advantage of hydrophobic IOLs in PCO prevention, we observed MJ14, a modified acrylic hydrophilic lens to carry the lowest incidence of YAG-CT due to visually disturbing PCO. Other hydrophilic IOLs (Asphina 409 M and MI60), however, were associated with the highest YAG-CT incidence with acrylic hydrophobic IOL (PCB00) being in the middle of the spectrum. As PCO is caused by remaining LECs migrating from anterior capsule and equator to the posterior capsule, a mechanical barrier preventing LEC migration can prevent PCO. One way to create such barrier is to make the posterior edge of the IOL optic sharp. To further increase its effectiveness, haptics can be angled forward in order to increase the pressure of the sharp posterior optic edge on the posterior capsule. In our study acrylic hydrophilic IOLs carry both lowest (MJ14) and highest (Asphina 409 M) YAG-CT rate, presumably due to differences in optic material, posterior optic edge sharpness, and haptic design. Additionally, MJ14 requires smaller incision size (1.4–1.8 mm) compared to other evaluated IOLs, potentially decreasing surgery-induced inflammatory response that contributes to PCO formation [26, 27]. Thus, the IOL material, optic edge design and haptic angulation together with incision size may all contribute to minimizing postoperative incidence of YAG-CT due to visually disturbing PCO. This finding stands in opposition to current belief that all hydrophobic acrylic IOLs are associated with lower PCO formation in comparison with all other IOLs, which is based on low-quality data [21]. Randomized clinical trials directly comparing one aspect of IOL design at a time as well as biomechanic studies in proper models are necessary to support further development of anti-PCO IOL design.

Another potential modifiable PCO risk factor is the type of tamponading agent used during phacovitrectomy. Rahman et al. have suggested that C2F6 tamponade carries almost three times higher risk of PCO compared to air or SF6 [9]. However, the authors did not adjust that observation for other factors, which may result in an incorrect conclusion due to the correlation of tamponade agent with underlying retinal disease. In our study, univariate analysis demonstrated that long-acting gas tamponade—SF6 decreased YAG-CT incidence more than two times compared to air tamponade. However, when corrected for other variables in the model, the effects of all tamponade types became statistically insignificant. Further studies are necessary to evaluate the role of tamponade on PCO development and YAG-CT rate, but it appears that there is no significant advantage of one agent over another.

Underlying retinal disease seems to be an important risk factor for PCO formation. Other conditions such as dry eye disease, glaucoma, age-related macular degeneration (AMD), hyperlipidemia and peptic ulcer disease have all been linked with increased PCO risk following cataract phacoemulsification surgery [28]. In our study, patients undergoing vitrectomy due to macular holes had the lowest YAG-CT incidence. Patients with rhegmatogenous retinal detachment, epiretinal membranes (ERM) and patients scheduled for removal of silicon oil following previous surgery had significantly increased YAG-CT incidence. Retinal detachment and the removal of silicon oil are generally associated with potent inflammatory response, ultimately resulting in activation of pro-fibrotic TGF-β pathway through IL-6, which likely causes PCO [29, 30]. Higher incidence of visually disturbing PCO development in ERM group might be caused by predisposition to scarring and fibroblast proliferation [31, 32]. Our findings suggest that patients, when scheduled for phacovitrectomy, should be informed about the risk PCO development and its association with their retinal disease. In high-risk patients, primary posterior capsulotomy may be considered [33, 34].

Diabetes has been associated with an increased risk of developing PCO following both cataract surgery and a combined procedure [35]. The exact mechanism by which diabetes influences PCO is not fully understood, but the current evidence indicates that chronic hyperglycemia leads to LEC activation. Specifically, high glucose levels lead to the formation of advanced glycation end products, activating protein kinase C signaling as well as inducing TGF-β and VEGF expression, all of which have been linked to the development of PCO [31, 36]. Our study confirms previous findings as type 2 diabetes was associated with a moderately increased YAG-CT incidence after phacovitrectomy.

Age is a recognized risk factor for PCO following cataract surgery, with a lower incidence seen in older patients. Several mechanisms may come into play, including an impaired regenerative capacity of LECs and changes in aqueous humor cytokine profile [37]. In our study age was inversely associated with YAG-CT incidence in univariate analysis, however after adjusting for other factors the results were not statistically significant (*p* = 0.13). We also found no significant effect of surgery duration on incidence of YAG-CT.

This study has several limitations. First, its retrospective design inherently increases the risk of confounding and bias compared with prospective analyses. Second, posterior capsule opacification was inferred from the occurrence of YAG-CT rather than from standardized, objective posterior capsule assessments; as the decision to perform YAG-CT may vary by surgeon judgment, patient-reported symptoms, and coexisting retinal pathology, the true PCO burden may be underestimated or overestimated, and vary between groups. Third, the distribution of intraocular lens types was markedly imbalanced, with one IOL model representing the majority of cases and the remaining lenses represented by substantially smaller subgroups, limiting the precision of comparisons between IOL types. Finally, capsulorrhexis diameter was not standardized beyond general surgical practice and varied modestly between 5 and 6 mm, which may influence anterior capsule–optic overlap and thus contribute to variability in PCO development unaccounted for in our analysis. To balance these limitations, we believe the long follow-up and multivariate analysis of over 10 potential risk factors are robust features of our study, providing preliminary insights into this issue.

In summary, we conclude that IOL choice is a key factor in decreasing visually disturbing PCO incidence in all subsets of patients, and that an IOL with hydrophilic acrylic material can be superior to hydrophobic acrylic in this respect. Overall, our study highlights the pressing need for properly designed randomized clinical trials comparing IOLs differing in only one aspect of the design at a time.

## Data Availability

No datasets were generated or analysed during the current study.
